# Dissociative paraplegia after epidural anesthesia: a case report

**DOI:** 10.1186/1752-1947-7-56

**Published:** 2013-02-27

**Authors:** Dusan Hirjak, Philipp A Thomann, Robert C Wolf, Norbert Weidner, Einar P Wilder-Smith

**Affiliations:** 1 Structural Neuroimaging Group, Department of General Psychiatry, Heidelberg University Hospital, Voßstraße 4, Heidelberg, D-69115, Germany; 2 Center for Psychosocial Medicine, Department of General Psychiatry, Heidelberg University Hospital, Heidelberg, Germany; 3 Spinal Cord Injury Center, Heidelberg University Hospital, Heidelberg, Germany; 4 Division of Neurology, National University Singapore, Singapore

**Keywords:** Dissociative paraplegia, Conversion disorder, Spinal cord injury, Psychiatry, Neurology

## Abstract

**Introduction:**

Clinicians are confronted with considerable difficulties in diagnosing conversion disorders such as dissociative paraplegia. In the literature, there is still no sufficient evidence regarding a typical pattern or general characteristics for this neuropsychiatric syndrome. Over the last decades case reports have described patients with similar personality traits, psychopathological characteristics, history and symptoms.

**Case presentation:**

We present the case of a 67-year-old Caucasian woman of high economic status and educational level with no psychopathological symptoms and no history of mental disorders who developed dissociative paraplegia after epidural anesthesia. The neurological examination revealed incongruous features, and repeated spine magnetic resonance imaging was normal. Three years earlier the patient had transient paralysis of her left lower limb without detectable cause.

**Conclusion:**

We identified an association between stressful life events and neurological anomalies. Crucial for the diagnosis of dissociative paraplegia is the neurological examination. Our case demonstrates that lack of psychopathological features and previous psychiatric diagnosis are not sufficient to exclude dissociative paraplegia. In patients with incongruous neurological findings and absent neurobiological correlates, clinicians should consider the presence of conversion disorders such as dissociative paraplegia.

## Introduction

Dissociative paraplegia (DP) is defined as an alteration or loss of function in the lower limbs without an anatomical or physiological explanation [1]. This type of illness belongs to the group of conversion disorders (CDs) and is rarely seen. The average prevalence of CDs in the general population is estimated at 5 to 22 per 100,000 persons [2,3]. Because it is rare, DP remains a diagnostic and therapeutic challenge on the borderline between neurology and psychiatry. When clinicians are confronted with uncommon patterns of paralysis, they are often obliged to embark on an exhausting diagnostic workup. Because of unfamiliarity with symptoms, lack of time, superficial or insufficient neurological and psychiatric examination, fear of missing an organic cause of paralysis, and deep-rooted prejudice against mental illnesses, DP may be underdiagnosed. Kanaan *et al*. [4] wrote that *“*today's neurologists once again face a disorder without an accepted model*”* [4, p. 961]. Even if clinicians strongly suspect DP, evidence-based practice guidelines for accurate assessment, treatment, and management of this borderline disorder are still lacking [5,6]. Moreover, the proportion of patients who present with evident psychopathological features or a full-blown psychiatric disorder is not known [7,8]. On the basis of reports in the literature, in the absence of mental state abnormalities, the diagnosis of DP becomes problematic for clinicians in all disciplines while treating patients with movement disorders [8].

Currently, there is still a gap between the diagnostic criteria for the dissociative disorders of the International Classification of Diseases, 10th revision (ICD-10), and the Diagnostic and Statistical Manual of Mental Disorders, Fourth Edition (DSM-IV), and what represents the typical clinical picture of a patient with DP [8]. According to ICD-10, the categories “conversion disorders” and “dissociative disorders” are used almost equivalently, which refer to a more psychodynamic approach [8,9]. This diagnostic concept includes, among others, dissociative amnesia, dissociative fugue, dissociative stupor, dissociative convulsion, dissociative anesthesia and sensory loss, and dissociative motor disorders [8,9]. Moreover, ICD-10 considers CDs as essentially acute-onset disorders with remission occurring within weeks or months, having an onset after stressful, traumatic, or intolerable events [9, p. E19]. In contrast, DSM-IV classifies CDs among the somatoform disorders, postulating long-term and chronic disorders including, among others, depersonalization disorder, dissociative amnesia, dissociative fugue, and dissociative identity disorder [8,9]. These diseases are generally rooted in the disrupted integration of perception, identity, memory, and consciousness [9, p. E19]. Such discrepancies in the definition of CDs might confound the diagnosis and delay appropriate treatment of patients with DP. Fortunately, in the preparation of ICD-11 and DSM-V, an intense debate about the current clinical picture, underlying mechanisms, and future criteria for CDs and DP is taking place [8].

Throughout the history of psychiatry and neurology, only a few cases of DP after epidural anesthesia have been reported [10-12]. These and other published case reports [1,4,13-18] described patients with characteristic features such as abrupt onset, female gender, young age, low socioeconomic status, low educational level, neurological disorders with incorrect anatomical pattern, bizarre movements, presence of evident psychological features such as traumatic experience, and a current or early diagnosis of a psychiatric disorder [2,3,13,14,16,19,20]. In this report, we describe the case of a patient with DP who presented after epidural anesthesia without the above-mentioned characteristic features.

## Case presentation

We have changed relevant personal information of the patient to protect her anonymity.

**Figure 1:**
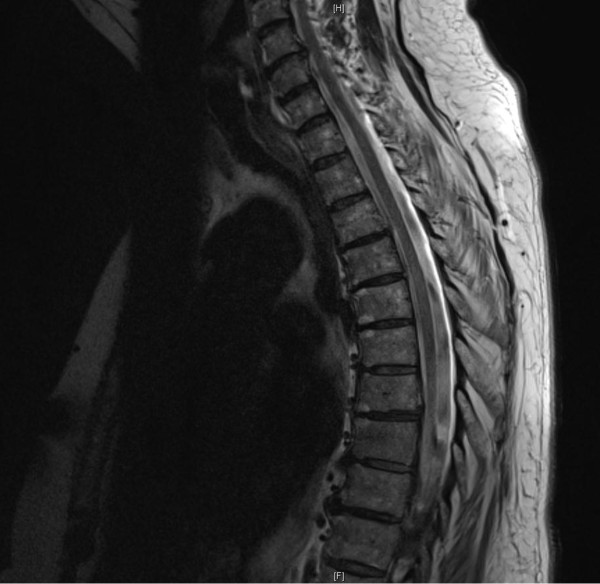
**Sagittal magnetic resonance imaging of the thoracic spine on day 1 after the abdominal surgery and epidural anesthesia.** Note the absence of canal compromise, edema, and bleeding.

**Figure 2:**
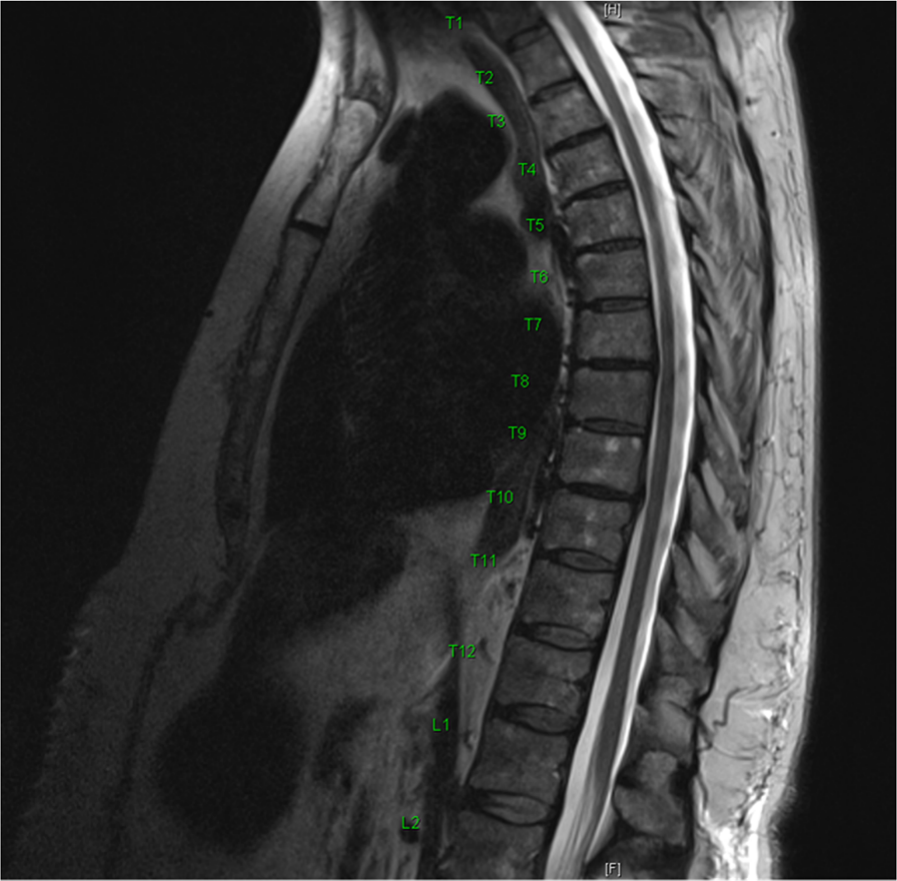
**Sagittal magnetic resonance imaging of the thoracolumbar spine on day 5 after the abdominal surgery and epidural anesthesia.** Note the absence of canal compromise, edema or bleeding.

Our patient was a 67-year-old Caucasian woman who was a pensioner with no history of neurological or psychiatric disorders. She had a college degree and had previously worked in social services. Before admission, she underwent abdominal surgery while under general anesthesia for extirpation of a pancreatic fistula with an uncomplicated epidural anesthetic consisting ropivacaine 0.2% and sufentanil 0.5μg/ml injected into the T7-T8 interspace. Before the surgery, the patient was informed about possible complications, including epidural bleeding, infection, and neurological disorders such as paraplegia. The pre-operative assessment was unremarkable. The patient tested negative for human immunodeficiency virus (HIV) and syphilis. The pre-operative neurological examination of the patient was not conducted in detail but as part of the general pre-operative examination, with motor and sensory functions documented as normal. During the entire procedure, no complications or major hemodynamic changes were recorded. Eight hours after the surgery, she complained of complete paralysis of both lower extremities and complete loss of sensation from dermatome T5 downward. A physical examination showed that her vital signs were stable. Post-operative neurological examination by a consultant neurologist on days 2 and 3 after surgery revealed loss of sensation affecting both lower limbs, abdomen, and chest, with a sensory level at the T5 dermatome and decreased strength of both lower extremities quantified as follows: hip flexion and extension bilaterally, 2/5 (Medical Research Council grading); knee extension, 2/5 (right) and 3/5 (left); knee flexion, 3–4/5 (right) and 3–4/5 (left); dorsiflexion, 4/5 (right) and 2/5 (left); plantar flexion, 4/5 (right) and 3/5 (left); and bilateral big toe extension, 4/5. Both lower limbs showed altering levels of increased muscle tone, ranging from mild to severely increased. Her knee jerks were brisk, and her Achilles tendon reflexes were difficult to elicit. Urinary retention occurred on days 2 and 3 after surgery. Rectal tone and sphincter function were weakened. The plantar response was equivocal on the right. With lifting of the legs into the air and subsequent release, the patient was able to tonically hold both legs suspended over the bed. Three magnetic resonance imaging (MRI) examinations on days 1, 2, and 5 after surgery failed to show any evidence of spinal cord, conus, cauda equina injury, edema, or bleeding (Figures 1 and 2). Motor-evoked as well as somatosensory-evoked potentials were normal. Extensive blood workup was normal. Because of the absence of edema or bleeding on all MRI scans and no local or systemic infection signs, the benefit of cerebrospinal fluid (CSF) analysis was deemed low and hence was not performed.

The mental state examination revealed a friendly person with appropriate general appearance and behavior. The patient demonstrated no cognitive or perceptual disorders. There was retained attention, concentration, and memory, along with a euthymic mood. Range of affect was appropriate without any abnormalities in the logic and coherence of thinking nor was there any evidence of disorders of thought content. Although she was initially concerned about her motor abilities and quality of life in the future, her diagnosis did not fulfill the ICD-10 or DSM-IV criteria for a major depressive disorder. She had no personal or family history of psychiatric disorders. Structured clinical interviews such as the Structured Clinical Interview for DSM I and II, the Beck Depression Inventory [21], the Hamilton Depression Rating Scale [22], the Brief Psychiatric Rating Scale [23], the Borderline Symptom List [24], the Difficulties in Emotion Regulation Scale [25], and the Dissociative Experiences Scale [26] were used to assess her current symptomatology. The patient showed no prominent signs or symptoms of axis I and II disorders. A diagnosis of DP was made according to ICD-10 criteria, based on the disparate neurological findings and the absence of somatic correlates for movement disability.

Intensive physical therapy was commenced 2 days after admission, and the patient’s symptoms began to resolve after the results of her tests and the potential psychological mechanism of the weakness were explained to her. She was discharged to a rehabilitation unit on day 14 after admission, with normal strength in both legs and ability to walk with assistance. During her outpatient presentation 3 months later, her gait was observed to have fully recovered and she was able to walk without any assistance.

## Discussion

The purpose of this case report is to highlight the phenomenon of DP. To the best of our knowledge, this report is only the fourth to describe a patient with DP after epidural anesthesia [10-12]. Acute paraplegia after epidural anesthesia is a serious emergency which can be caused by chemical, physical, or ischemic factors affecting the spinal cord [11]. Diagnostic work-up is necessary to exclude hematoma, abscess, inflammation, and transient ischemic attack (TIA) of the spinal cord. In some patients, minor bleeding or an evolving lesion may not manifest for a further 2 to 3 days. For this reason, we obtained three sequential MRI studies of the whole spinal cord over a 5-day period. Regarding the difficulty of eliciting Achilles tendon reflexes and the loss of bladder and bowel control, it should be noted that the Achilles tendon reflex in particular can be difficult to elicit by less experienced clinicians. When examined in our hospital several days later, these parameters were normal. It is also important to be aware that anomalies of neurological reflexes and loss of rectal tone may occur in CDs [17,20]. No clear-cut explanation for this has been offered, though everyday clinical experience suggests that outcome parameters of tested reflexes are operator-dependent, with patient relaxation playing an important role. A further possible contributor may be the remaining influence of ropivacaine and sufentanil on the first day after anesthesia. However, because of the short half-life of both anesthetics, and lacking evidence for a release of local anesthetics into the CSF after patient movement, transient paraplegia after spinal anesthesia was unlikely in our patient. Moreover, since no CSF or blood was aspirated at any point during catheter placement, an injury of vascular supply, intravascular instillation, intrathecal leakage through the dural puncture, or a spinal cord TIA were ruled out. The fact that the patient was able to hold both legs suspended over the bed while not being able to exert other forms of muscle power into the lower limbs raised the suspicion of a non-organic etiology.

Biographically, we found some overlap between neurological symptoms and stressful life events. In 1995, the patient had problems with her mother, who lived in a nursing home. Her mother blamed the family, in particular her daughter, for having to live in a nursing home, for which the patient felt stressed and guilty. After the death of her mother in 1995, she underwent 2 years of sporadic supportive counseling by her general practitioner. According to the patient’s medical records and her narratives, she did not meet the criteria for a psychiatric diagnosis according to ICD-10 or DSM-IV. In 2009, 3 weeks after the death of her husband, she developed monoplegia of her left lower extremity and abasia of unknown origin. A neurologist found no cause for her movement disorder. Three weeks later she was fully recovered.

A parallel between both stressful and traumatic life events in 1995 and 2009 and her current health problems was identified. In 1995, the crisis and possible development of neurological symptoms were intercepted through supportive counseling. In 2009, the patient had difficulty coping with her husband’s death. She did not receive any psychotherapeutic treatment at that time and dealt with the loss on her own. We speculate that the movement disorder might represent a coping mechanism for the loss of her husband. Similarly, the surgery and epidural anesthesia in 2012 might be considered a trigger for her developing DP [27]. However, we still have no evidence regarding possible traumatic experiences as a cause for her development of DP [28]. A possible explanation might be the subjective experience of pre-operative patient information concerning the epidural catheter and the possible complications after epidural anesthesia. This “angst”-laden information might lead to a desperate situation and trigger or reactivate earlier existential fears or memories. In fact, an epidural catheter and numbness of both lower limbs seems to be a possible learning model for patients to develop a DP, because of a life-threatening experience and being at the mercy of both physicians and technical devices in the operating room. In our case, both matters can also be considered as traumatic because of their uncontrollable character. Moreover, the patient’s perception of the anatomical location of the epidural anesthesia with the potential for direct trauma through the needle (at T7 and T8) as well as the pancreatic surgery itself might have initiated the observed neurological dysfunctions. However, the possible traumatic locus in comparison to the vascular supply of the spinal cord in that area is not anatomically consistent with the neurological findings detected. In addition, the patient’s three MRI scans showed no signs of bleeding or inflammation (Figures 1 and 2). Fortunately, after the neurological findings and psychological mechanisms underlying both movement disorders were discussed with the patient, she showed good insight into the reasons for her DP and her condition improved rapidly.

In patients with CDs such as DP, a high rate of psychiatric co-morbidity has been identified. The majority of the patients have somatoform disorder, generalized anxiety disorder, dysthymic disorder, simple phobia, obsessive-compulsive disorder, and major depression [29-31]. In our patient, however, structured clinical interviews did not identify any acute or chronic psychiatric co-morbidity. The patient presented with an entirely typical appearance with no psychopathological features. In particular, we found no indication of co-morbid dissociative disorders, feigning, la belle indifference, or alexithymia. Our observations support the view of Stone *et al*. [32], Chabrol *et al*. [33], and Kanaan *et al*. [8], who agreed that acute or chronic psychopathological symptoms are not pathognomonic for CDs such as DP.

Furthermore, the case of our patient demonstrates that individuals presenting with DP might be older, sophisticated, well-educated, and of high economic status. When diagnosing DP, neuroimaging and electrophysiological examination are obligatory and helpful in excluding organic paralysis. Even if clinicians with little experience might fear missing an organic cause of the presenting symptom, DP should not be considered solely as a diagnosis of exclusion. Moreover, after a careful assessment of the patient’s case history and rigorous physical assessment, neuroimaging or electrophysiological testing for structural diagnoses may not be warranted or as extensive if the neurological examination is not consistent with anatomical localization of a structural lesion. To prevent disease chronification, some authors suggest that patients should be informed early about the diagnosis and treated subsequently [6]. Therefore, it is necessary to conduct multifunctional approach therapy, including psychotherapy, relaxation techniques, autogenic training, and intensive physical therapy. Physical therapy especially might help the patient to give up symptoms without losing face and consequently improve bodily experience, movement ability, interpersonal attunement, and social well being. There is also some evidence suggesting that patient education regarding the underlying mechanisms is helpful [6], but special attention must be paid to possible stigmatization and labeling of the patient. Emphasis should be placed on the reversibility and good prognosis of the disorder, as was the case for our patient.

## Conclusion

Even when psychopathological features or serious mental health problems are missing, CDs such as DP should be considered, even early in the diagnostic process. Elicitation of incongruous or unusual neurological findings should be followed up by careful examination for non-transparent previous diagnostic tests and unexpected somatic reactions to life events, because the wealth of possible triggers makes DP special and problematic for all involved disciplines.

## Consent

Written informed consent was obtained from the patient for publication of this case report and any accompanying images. A copy of the written consent is available for review by the Editor-in-Chief of this journal.

## Competing interests

The authors declare that they have no competing interests.

## Authors’ contributions

DH and PAT analyzed and interpreted the patient data regarding the mental state examination. NW and EWS performed the neurological examination of the patient. DH and EWS wrote the manuscript. DH and RCW were involved in the revision of the manuscript. All authors read and approved the final manuscript.
